# Types of Inheritance and Genes Associated with Familial Meniere Disease

**DOI:** 10.1007/s10162-023-00896-0

**Published:** 2023-04-06

**Authors:** Alberto M. Parra-Perez, Jose A. Lopez-Escamez

**Affiliations:** 1 Meniere’s Disease Neuroscience Research Program, Faculty of Medicine & Health, School of Medical Sciences, The Kolling Institute, University of Sydney, 10 Westbourne St, St Leonards NSW 2064, Sydney, NSW Australia; 2 Otology and Neurotology Group CTS495, Department of Genomic Medicine, GENYO - Centre for Genomics and Oncological Research - Pfizer, University of Granada, PTS, Junta de Andalucía, Granada, Spain; 3 Division of Otolaryngology, Department of Surgery, Instituto de Investigación Biosanitaria, Ibs.GRANADA, Universidad de Granada, Granada, Spain; 4 Sensorineural Pathology Programme, Centro de Investigación Biomédica en Red en Enfermedades Raras, CIBERER, Madrid, Spain

**Keywords:** Meniere’s disease, Hearing loss, Exome sequencing, Genetic, Genomics, Tectorial membrane, Otolithic membrane, OTOG gene, MYO7A gene, TECTA gene

## Abstract

Meniere disease (MD) is a rare disorder of the inner ear defined by sensorineural hearing loss (SNHL) associated with episodes of vertigo and tinnitus. The phenotype is variable, and it may be associated with other comorbidities such as migraine, respiratory allergies, and several autoimmune disorders. The condition has a significant heritability according to epidemiological and familial segregation studies. Familial MD is found in 10% of cases, the most frequently found genes being *OTOG*, *MYO7A*, and *TECTA*, previously associated with autosomal dominant and recessive non-syndromic SNHL. These findings suggest a new hypothesis where proteins involved in the extracellular structures in the apical surface of sensory epithelia (otolithic and tectorial membranes) and proteins in the stereocilia links would be key elements in the pathophysiology of MD. The ionic homeostasis of the otolithic and tectorial membranes could be critical to suppress the innate motility of individual hair cell bundles. Initially, focal detachment of these extracellular membranes may cause random depolarization of hair cells and will explain changes in tinnitus loudness or trigger vertigo attacks in early stages of MD. With the progression of the disease, a larger detachment will lead to an otolithic membrane herniation into the horizontal semicircular canal with dissociation in caloric and head impulse responses. Familial MD shows different types of inheritance, including autosomal dominant and compound recessive patterns and implementation of genetic testing will improve our understanding of the genetic structure of MD.

## Introduction

Meniere disease (MD) is a term used to describe patients with an audio-vestibular phenotype that includes episodes of vertigo associated with variable aural symptoms (hearing loss, tinnitus, and aural pressure) [1]. The phenotype is not limited to the inner ear, and it may be associated with other comorbidities such as migraine, allergic rhinitis, asthma, and several autoimmune or autoinflammatory disorders [1–4]. Most of the episodes are reported as spontaneous, but some patients report that a loud noise may trigger vestibular symptoms [5]. There is a great variability in the onset of the symptoms [6], and many patients initially show a partial syndrome [7]. This clinical heterogeneity makes the diagnosis a challenge in the first few years, since no biological marker is available to define MD [8].

The diagnostic criteria were initially proposed by the Japanese Ménière’s disease study group in 1974. The American Academy of Otolaryngology-Head and Neck Surgery (AAO-HNS) developed guidelines for diagnosis and therapy evaluation of MD in 1972 and revised them in 1985 and 1995 [9]. In this century, the criteria were redefined by the Classification Committee for Vestibular Disorders of the Bárány Society, the Japan Society for Equilibrium Research, the European Academy of Otology and Neurotology (EAONO), the Equilibrium Committee of the American Academy of Otolaryngology-Head and Neck Surgery (AAO-HNS), and the Korean Balance Society in 2015 [10]. These criteria improved the clinical diagnosis by excluding patients with conductive hearing loss or isolated high-frequency sensorineural hearing loss (SNHL) and may include several comorbidities such as allergy, migraine, or autoimmune diseases in the condition [8].

Histopathological studies in human temporal bones have consistently shown an accumulation of endolymph, termed endolymphatic hydrops, in the vestibular end organs (saccule and utricle) and the cochlear duct in most patients with MD [11, 12]. This finding probably reflects a histopathological damage in the inner ear, since it has also been found in other patients with SNHL > 50 dB without episodes of vertigo [13] or patients with vestibular migraine [14].

Two major hypotheses are currently accepted to explain the pathophysiology of MD: a chronic autoinflammatory process defined by a immune dysfunction with high levels of several cytokines and chemokines [15, 16], and rare allelic variants in coding regions reported in several genes, many of them previously associated with non-syndromic SNHL [17]. Although there have been considerable advances in the last 10 years, the contribution of genetic factors to the occurrence of MD symptoms is not yet fully understood.

The purpose of this review is to summarize the evidence that supports the genetic contribution to MD, including familial aggregation and exome sequencing studies. Furthermore, we describe the main genes reported in multiplex familial MD and elaborate a hypothesis regarding the potential role of stereocilia, otolithic membrane (OM), and tectorial membrane (TM) proteins in MD. For this, we conducted a Pubmed search with the following keywords: (familial [Title/Abstract] OR family [Title/Abstract] OR gene [Title/Abstract] OR genes [Title/Abstract] OR inheritance [Title/Abstract] OR variation [Title/Abstract] OR mutation [Title/Abstract]) AND (meniere disease [Title/Abstract] OR meniere’s disease [Title/Abstract]). The search was filtered by the last 22 years (2000–2022) and limited to publication written in English, including original and review papers.

## Familial MD

Madeleine Ray Brown was the first that reported two families with MD in 1941. The first family consisted of two sisters and a brother in one French Canadian consanguineous family with paroxysmal vertigo associated with sensorineural hearing loss. All started with tinnitus before the vertigo attacks and the age of onset were 46, 32, and 35 respectively [18]. In the second family, two identical twins showed audio-vestibular symptoms; one reported a sudden increase of deafness, tinnitus, and paroxysmal attacks of vertigo since he was 31 years old; the second only showed a non-progressive hearing loss [18].

Bernstein described in 1965 seven families in which more than one member had episodic vertigo or hearing loss. Two families had histories of allergy and members of three other families were suffering from migraine headaches [19]. Although some of these patients were partial syndromes and cannot be defined as MD, these early studies started to define clinical subgroups of MD patients that have been confirmed in large MD cohorts [8].

Morrison et al. reported a series of 46 British families with MD. Most of these families showed an autosomal dominant inheritance with reduced penetrance [20]. Anticipation was also observed, although this could be a bias of the search strategy. In this set of British families, maternal transmission was more frequent than paternal inheritance.

Familial clustering has been reported in about 9% of cases in Spanish population [21], and in 6% of cases in South Korea [22]. The sibling recurrence risk ratio for MD that estimates the odds to develop MD if the proband has a first degree relative with MD compared to the prevalence in the general population was 16–48 [21].

Most MD patients do not report relatives with the same clinical picture; however, it is not uncommon to find relatives with SNHL or episodic vertigo that have not been investigated in detail and could be partial syndromes [23]. For this reason, most cases are considered sporadic, but familial MD (FMD) has been repeatedly described in European descendent population in 5–20% of cases [24].

Several types of inheritance have been reported in FMD, including autosomal dominant (AD) and autosomal recessive (AR) inheritance [18, 25]. Moreover, digenic and multiallelic inheritance have also been found in FMD [26]. These findings start to define a complex inheritance that combined with some environmental triggers may result in a familial disorder with variable expressivity [23] that it is observed even in the same family (i.e., uni/bilateral hearing loss, early/late age of onset).

## Exome Sequencing Studies in Familial MD

The application of exome sequencing technology to the diagnosis of MD has contributed to decipher the genetics underpinnings of familial MD [24, 25]. Several rare mutations and target genes have been reported in different families with MD in Spain, South Korea, Finland, Sweden, and Iran [27]. The first Spanish family was reported by Requena et al. in 2015 [28]. The family consisted of three women with the complete MD phenotype over three generations which segregated two heterozygous rare variants in *FAM136A* and *DTNA* genes which were classified as pathogenic [28].

The first variant was a nonsense novel variant in the *FAM136A* gene leading to a stop codon (GRCh38 chr2:70300842G > A; NM_032822.3); the second variant was an ultrarare missense heterozygous variant found in the *DTNA* gene (GRCh38 chr18: 34882130G > T; NM_001390.5) that results in a p.Val715Phe substitution and generates a novel splice-site sequence predicted as a constitutive acceptor [28].

Thirteen genes have been associated with AD or AR familial MD. Intriguingly, each of these genes has a different function within the inner ear ranging from playing a role in the cytoskeleton structure of cochlear hair cells, the stress oxidative to axonal guidance pathways [29]. The main criticism on these familial studies is that most of the reported variants were only found in one family, and it cannot be ruled out that these mutations were private mutations restricted to each one of these families. For this reason, additional families with pathogenic or likely pathogenic variants in the same gene segregating the phenotype are needed to support the association between these candidate genes and FMD, according to the criteria for variant prioritization of the American College of Medical Genetics modified for hearing loss genes [30].

However, this issue was solved by Roman-Naranjo et al. in 2020, with the report of 6 Spanish families segregating 2 missense variants in the *OTOG* gene [25]. Two heterozygous variants of unknown significance (VUS), chr11:17557227G > A and chr11:17611374C > T (GRCh38; NM_001292063.2), were found in four unrelated patients from four different families with MD. Moreover, another heterozygous variant (chr11:17553211G > A), classified as pathogenic, was observed in two MD cases from another two unrelated families; both families also shared a novel variant chr11:17573200G > A, and one of them also had a third variant, chr11:17606001G > A. Although a double *Otog* mutant mouse will be needed to confirm the functional effect of these VUS, these findings support that *OTOG* gene is associated with heterozygous compound recessive inheritance in the 6 families [25].

**Fig. 1:**
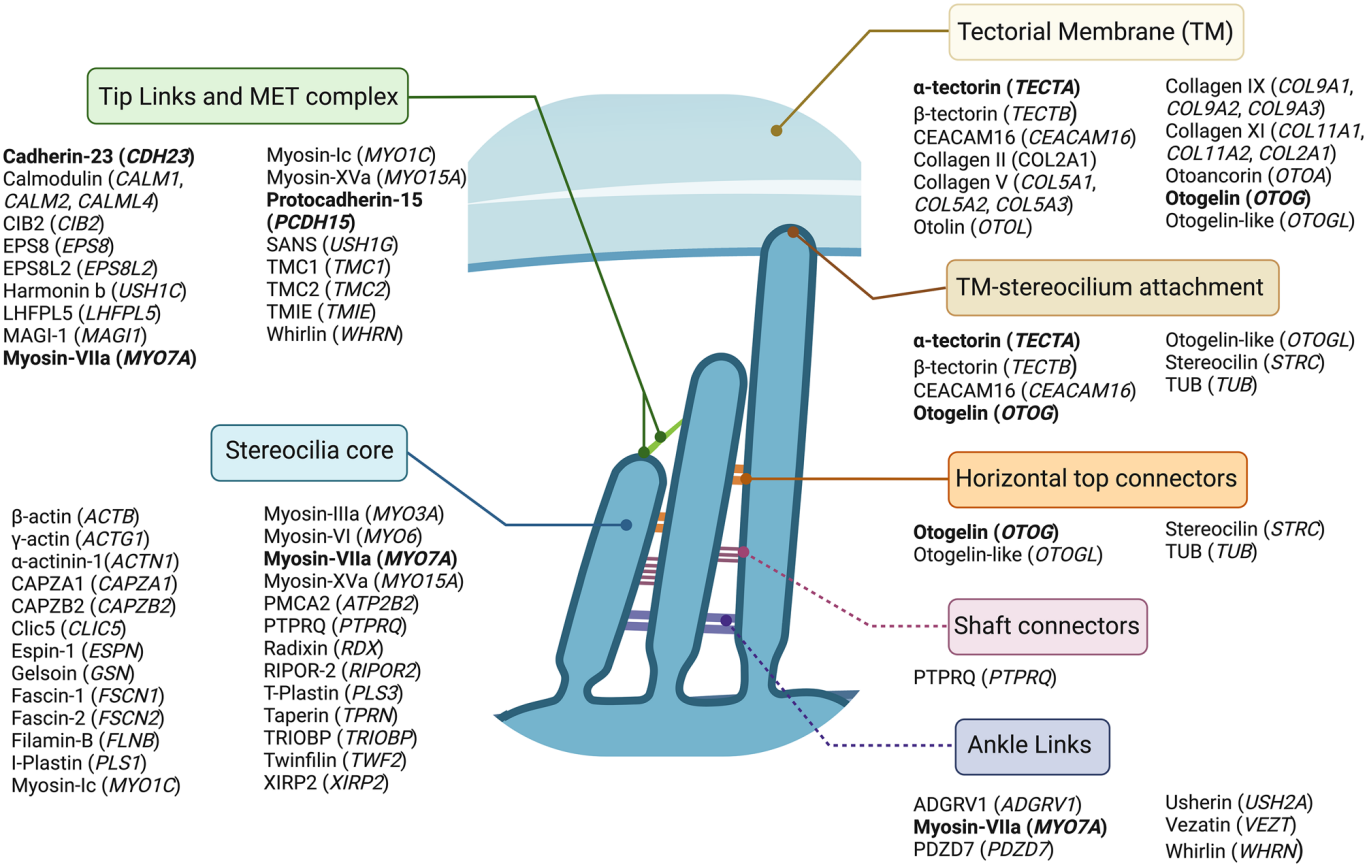
Schematic representation of the mammalian cochlear hair cell stereocilia. Proteins and their coding genes are listed for the stereocilia links (tip links, horizontal top connectors, shaft connectors, and ankle links) and between the stereocilia and the tectorial membrane. In addition, proteins that are part of the mechanoelectrical transduction (MET) complex [61], the tectorial membrane [62], and some of the most relevant proteins that constitute the stereocilium are indicated [63, 64]. Mutated structural proteins in familial Meniere disease are indicated in bold. Ankle links and shaft connectors are not found in mature hair cells, although they are found during hair cell development. ADGRV1: adhesion G-protein coupled receptor V1; CAPZA1: Capping Actin Protein of Muscle Z-Line Subunit Alpha 1; CAPZB2: Capping Actin Protein of Muscle Z-Line Subunit Beta 2; CEACAM16: carcinoembryonic antigen-related cell adhesion molecule 16; CIB2: calcium and integrin-binding family member 2; Clic5: chloride intracellular channel protein 5; EPS8: epidermal growth factor receptor kinase substrate 8; EPS8L2: epidermal growth factor receptor kinase substrate 8-like protein 2; LHFPL5: LHFPL (lipoma HMGIC fusion partner-like) tetraspan subfamily member 5 protein; MAGI-1: membrane-associated guanylate kinase, WW and PDZ domain-containing protein 1; PDZD7: PDZ domain-containing protein 7; PMCA2: plasma membrane calcium-transporting ATPase 2; PTPRQ Protein Tyrosine Phosphatase Receptor Type Q; RIPOR2: RHO Family Interacting Cell Polarization Regulator 2; SANS: pre-mRNA splicing regulator USH1G; TMIE: transmembrane inner ear expressed protein; TMC1/2: Transmembrane channel-like protein 1 and 2 dimer; TRIOBP: TRIO And F-Actin Binding Protein; TUB: tubby protein homolog; XIRP2: Xin actin-binding repeat-containing protein 2. Figure created with BioRender.com

Multiple MD families carrying rare variants in genes encoding proteins involved in the architecture of the hair cells stereocilia and their attachment to the TM have been found [31]. Roman-Naranjo et al. found co-segregation in several novels and rare variants in the *MYO7A* gene with other genes including *CDH23*, *PCDH15*, and *ADGRV1* involved in the mechanoelectric transduction (MET) complex and the interciliary links of the hair cells in several MD families, suggesting a digenic inheritance model [31] (Fig. 1).

*CDH23* and *PCDH15* genes encode for cadherin-23 and protocadherin-15, two calcium-dependent cell adhesion proteins that show a direct interaction in the apical surface of hair cells to form the tip links between stereocilia. These links are required for maintaining the proper organization of the stereocilia bundle of hair cells in the sensory epithelia of the organ of Corti and the vestibular organs during embryonic and early postnatal development [32]. Furthermore, cadherin-23 and protocadherin-15 mediate SNHL and Usher syndrome type 1 by digenic recessive inheritance [32]. They are part of the functional gene network formed by *USH1C* (harmonin b), *USH1G* (*SANS*), *CDH23*, and *MYO7A* (myosin-VIIa) that regulate MET in cochlear hair cells [33, 34].

Moreover, by using a gene burden analysis and applying multiallelic inheritance models in SNHL genes, enrichment of rare missense variants in the *OTOG* gene were found in 15 families with MD suggesting multiallelic inheritance, including the 6 families previously mentioned with compound recessive inheritance [25]. Finally, the presence of rare missense variants and frameshift deletions in the *TECTA* gene within 6 MD families suggests a role of this gene in the pathophysiology of the disease [35].

Although familial MD has been associated to several genes, the incomplete penetrance and variable expressivity within families remain unexplained and the role of regulatory elements (promoters, enhancers, non-coding RNA species) in the MD phenotype deserves further research. Several microRNAs and other non-coding elements are known to be associated with SNHL and they are candidate targets for therapy [36]. An epigenetic study was performed using whole-genome bisulfite sequencing (WGBS) suggesting that the DNA methylation signature could allow distinguishing between MD patients and controls [37]. In this study, a great number of differentially methylated CpGs were found when comparing MD patients to controls. Of note, few of these CpGs involved several hearing loss genes, including *CDH23*, *PCDH15*, or *ADGRV1,* that encode for stereocilia link proteins [37]; however, the study was performed in a small group of sporadic patients with MD and further studies in familial cases are needed to clarify the role of methylated CpGs in these genes.

All these studies point to a complex inheritance model, including digenic and multiallelic inheritance. Genes encoding proteins linking the hair cells stereocilia in the sensory epithelia and proteins in the TM and OM should be considered potential molecular targets associated with the onset of FMD.

## Types of Inheritance and Genes in MD

### Autosomal Dominant MD

Nine genes including *FAM136A*, *DTNA*, *PRKCB*, *COCH*, *DPT, SEMA3D*, *TECTA*, *GUSB*, and *SLC6A7* have been reported in autosomal dominant familial MD (Table 1) [27, 35]. However, *TECTA* gene is the only gene that have been involved in 2 unrelated families with autosomal dominant MD [35].

**Table 1:** Genes and variants reported in autosomal dominant familial MD

**Gene**	**Chr**	**Position**^**a**^	**ID**	**cDNA**	**Protein**	**Variant effect**	**Allelic frequency**^**b**^		**ACMG classification**	**CADD score**
							**gnomAD**	**Other**		
*FAM136A*	2	70300842	rs690016537	NM_032822.3:c.226C >T	p.Gln76*	Nonsense	Novel		Pathogenic (PS3, PS4, PM2, PM4, PP3)	41.0
*DTNA*	18	34882130	rs533568822	NM_001390.5:c.2143G >T	p.Val715Phe	Missense	1.32 × 10^–5^	NF(CSVS) 2.5 × 10^–5^ (ExAC)	Pathogenic (PS3, PS4, BP1)	24.9
*PRKCB*	16	23988577	rs1131692056	NM_212535.3:c.275G >T	p.Gly92Val	Missense^c^	6.57 × 10^–6^	NF(CSVS)	Likely pathogenic (PS4, PM2, PP3, PP5)	28.2
*COCH*	14	30880590	-	NM_004086.2:c.485G >A	p.Cys162Tyr	Missense^c^	Novel		Likely pathogenic (PS4, PM2, PP2, PP3, PP5)	28.1
*DPT*	1	168696611	rs748718975	NM_001937.5:c.544C >T	p.Arg182Cys	Missense^c^	1.31 × 10^–5^	NF(CSVS) 2.5 × 10^–5^ (ExAC)	Likely pathogenic (PS4, PM1, PP3, PP5, BP1)	32.0
*SEMA3D*	7	85012812	rs1057519374	NM_001384900.1:c.1738C >T	p.Pro580Ser	Missense^c^	6.59 × 10^–6^	NF(CSVS)	Pathogenic (PS4, PM1, PM2, PP3, PP5)	24.5
*TECTA*	11	121152980	rs774697277	NM_005422.4:c.4205G >C	p.Cys1402Ser	Missense^d^	3.33 × 10^–5^	10^–3^ (CSVS) 3.3 × 10^–5^ (ExAC)	Uncertain significance (PP3, PM2, PP1)	28.0
	11	121157956	-	NM_005422.4:c.4422delC	p.Asn1474LysfsTer91	Frameshift^d^	Novel		Likely pathogenic (PM2, PVS1)	-
	11	121158016	rs200544452	NM_005422.4:c.4481 T >C	p.Val1494Ala	Missense^c, d^	6.57 × 10^–5^	10^–3^(CSVS) 5.9 × 10^–5^ (ExAC)	Uncertain significance (BP4, PM2, PS3, PP1, PP4)	24.3
	11	121165368	-	NM_005422.4:c.5368C >T	p.Pro1790Ser	Missense^c, d^	Novel		Uncertain significance (BP4, PM2, PP4)	16.1
	11	121189864	rs1223512271	NM_005422.4:c.6353delG	p.Gly2118ProfsTer22	Frameshift^c, d^	6.58 × 10^–6^	NF(CSVS)	Likely pathogenic (PM2, PM4, PP1, PP5)	35.0
*GUSB*	9	65980297	rs1268678201	NM_000181.4:c.323C >T	p.Pro108Leu	Missense^c^	1.99 × 10^–5^	NF(CSVS) 1.92 × 10^–4^ (gnomAD Finn)	Uncertain significance (PM2, PP1, PP3)	25.3
*SLC6A7*	5	150196820	rs775035174	NM_014228.5:c.322G >C	p.Val108Leu	Missense	1.25 × 10^–4^	NF(CSVS) 6.6 × 10^–5^ (ExAC) 1.69 × 10^–3^ (gnomAD Finn)	Uncertain significance (BP4, PM2, PP4)	24.5

*ACMG* American College of Medical Genetics and Genomics, *AD* autosomal dominant inheritance pattern, *CADD* Combined Annotation Dependent Depletion, *CSVS* Collaborative Spanish Variant Server, *ExAC* Exome Aggregation Consortium, *gnomAD* Genome Aggregation Database, *gnomAD Finn* Genome Aggregation Database Finnish Population, *ID* reference Single Nucleotide Polymorphism identifier, *NF* not found

*stop codon

^a^Positions have been updated according to the GRCh38/hg38 reference genome

^b^allelic frequencies reported in the original reports have been updated according to the available information in the last version of the reference database (gnomAD v3.1.2)

^c^incomplete penetrance

^d^multiple inheritance

In this study, Roman-Naranjo et al. have reported 6 families with rare missense and frameshift variants [35]. The variant p.Val1494Ala was found in two families and one sporadic case and with a minor allelic frequency 8.8 × 10^–5^ in Non-Finish European was classified as VUS. Further studies are needed to clarify the functional effect of this variant.

Of note, two of the 6 families showed two heterozygous frameshift deletions that were classified as likely pathogenic (p.Asn1474LysfsTer91 in exon 14 and p.Gly2118ProfsTer22 in exon 23, respectively). Both deletions generate a shorter α-tectorin with a modified C‐terminal region that involves the glycosylphosphatidylinositol (GPI) anchorage signal. This signal peptide is essential to prevent diffusion of secreted TM proteins, and these deletions lead to a random aggregation of collagen fibrils and thinner TM with low tolerance to changes in endolymphatic pressure [38].

*COCH* is the causal gene for DFNA9, characterized by a progressive high-frequency SNHL with variable progressive vestibular impairment [39]; however, a family was reported in South Korea with episodic vertigo and bilateral SNHL with the mutation p.Cys162Tyr was considered a MD-like phenotype [40]. In the adult mouse cochlea, Cochlin is also a protein expressed in the fibrocytes of spiral ligament and spiral limbus, but not in the organ of Corti or the stria vascularis. In the vestibular, cristae show intense staining in the fibrocytes and stroma underlying the sensorineural epithelium and the ampullary wall [41].

*DTNA* encodes α-dystrobrevin, a structural component of the dystrophin-glycoprotein complex, leading to progressive brain oedema in the knockout mouse [42]. Evidence to involve α-dystrobrevin in familial MD have been from a Drosophila model [43]. Requena et al. (2022) have investigated two Drosophila homologues, Dystrobrevin (Dyb) and Dystrophin (Dys), in Johnston’s Organ function, showing that Dyb mutant flies exhibit defects in proprioception and early onset hearing loss caused by a progressive loss MET associated with a reduction of sensitive transducers [43].

However, for the seven remaining genes, pathogenicity is based on bioinformatic predictors and additional evidence from new families segregating rare variants in these genes are required to support the association.

### Autosomal Recessive MD

Table 2 list the 4 genes that have been reported in AR familial MD, including *HMX2*, *LSAMP*, *OTOG*, and *STRC.*

**Table 2:** Genes and variants reported in autosomal recessive familial MD

**Gene**	**Chr**	**Position**^**a**^	**ID**	**cDNA**	**Protein**	**Variant effect**	**Allelic frequency**^**b**^		**ACMG classification**	**CADD score**
							**gnomAD**	**Other**		
*STRC*	15	43604750	rs144948296	NM_153700.2:c.4027C >T	p.Gln1343*	Nonsense	1.97 × 10^–5^	NF (CSVS)	Pathogenic	40.00
								3.43 × 10^–4^ (ExAC)	(PSV1, PS4, PM2, PP3, PP5)	
*HMX2*	10	123150118	rs1274867386	NM_005519.2:c.817 T >A	p.Tyr273Asn	Missense^d^	6.57 × 10^–6^	NF (CSVS)	Likely pathogenic	31.00
									(PS4, PM2, PP3)	
*TMEM55*	14	20459211	rs201529818	NM_001100814.3:c.706C >T	p.Leu229Phe	Missense^d^	9.56 × 10^–4^	NF (CSVS)	Uncertain significance	25.80
*(PIP4P1)*								8.2 × 10^–5^ (ExAC)	(PS4, PP3, BS1)	
*OTOG*	11	17553211	rs552304627	NM_001292063.2:c.421G >A	p.Val141Met	Missense^d^	8.35 × 10^–4^	4.1 × 10^–4^ (ExAC)	Pathogenic	33.00
								4 × 10^–3^ (CSVS)	(PVS1, PS4, PM2, PP3, BP1)	
	11	17557227	rs61978648	NM_001292063.2:c.805G >A	p.Val269Ile	Missense^c, d^	2.04 × 10^–2^	1.4 × 10^–2^ (CSVS)	Likely benign	19.12
								8.01 × 10^–3^ (ExAC)	(PS4, BP1, BP4, BP6)	
	11	17573200	-	NM_001292063.2:c.2203C >A	p.Pro747Thr	Missense	Novel		Uncertain significance	21.90
									(PS4, PM2, BP1, BP4)	
	11	17599671	rs117005078	NM_001292063.2:c.3719C >T	p.Pro1240Leu	Missense^d^	3.3 × 10^–3^	4 × 10^–3^ (CSVS)	Likely pathogenic	33.00
								1.68 × 10^–3^ (ExAC)	(PS4, PM2, PP3, BP1)	
	11	17606001	rs145689709	NM_001292063.2:c.4058G >A	p.Arg1353Gln	Missense^c, d^	2.84 × 10^–3^	6 × 10^–3^ (CSVS)	Uncertain significance	22.00
								1.98 × 10^–3^ (ExAC)	(PS4, PM2, BP1, BP4, BP6)	
	11	17609906	rs117380920	NM_001292063.2:c.4642C >T	p.Leu1548Phe	Missense^c^	8 × 10^–3^	1.3 × 10^–2^ (CSVS)	Benign	12.42
								1.07 × 10^–2^ (ExAC)	(PS4, BS1, BS2, BP1, BP4, BP6)	
	11	17611374	rs61736002	NM_001292063.2:c.6110C >T	p.Ala2037Val	Missense^d^	2.41 × 10^–3^	4 × 10^–3^ (CSVS)	Uncertain significance	7.61
								3.74 × 10^–3^ (ExAC)	(PS4, PM2, BP1, BP4)	
	11	17635125	rs76461792	NM_001292063.2:c.7667G >A	p.Arg2556Gln	Missense^d^	3.06 × 10^–3^	4 × 10^–3^ (CSVS)	Benign	23.50
								2.95 × 10^–3^ (ExAC)	(PS4, BS1, BS2, BP1, BP4, BP6)	
	11	17642200	rs117315845	NM_001292063.2:c.8405G >A	p.Arg2802His	Missense^c, d^	2.04 × 10^–3^	6 × 10^–3^ (CSVS)	Uncertain significance	16.79
								3.68 × 10^–3^ (ExAC)	(PS4, PM2, BP1, BP4, BP6)	
	11	17645592	rs61997203	NM_001292063.2:c.8526G >C	p.Lys2842Asn	Missense	1.57 × 10^–2^	1.9 × 10^–2^ (CSVS)	Benign	24.20
								9.79 × 10^–3^ (ExAC)	(PS4, BS1, BS2, BP1, BP6)	
*LSAMP*	3	115842555	-	NM_001318915.2:c.673 T >C	p. Lys225Glu	Missense	Novel		Likely pathogenic	25.90
									(PS4, PM2)	

*ACMG* American College of Medical Genetics and Genomics, *AR* autosomal recessive inheritance pattern, *CADD* Combined Annotation Dependent Depletion, *CSVS* Collaborative Spanish Variant Server, *ExAC* Exome Aggregation Consortium, *gnomAD* Genome Aggregation Database, *ID* reference Single Nucleotide Polymorphism identifier, *NF* not found

*stop codon

^a^Positions have been updated according to the GRCh38/hg38 reference genome

^b^allelic frequencies reported in the original reports have been updated according to the available information in the last version of the reference database (gnomAD v3.1.2)

^c^incomplete penetrance

^d^multiple inheritance

The most relevant gene in familial MD is *OTOG* which encodes for otogelin Spanish families have compound recessive inheritance in 6% of the cases, but rare VUS or likely pathogenic variants are found in 15% of the families [25].

Otogelin is a secreted protein related to epithelial mucins required for the anchoring of the OM to the hair cell stereocilia in the sensory epithelia in the vestibule and the organ of Corti [44]. It is involved in the organization and stabilization of the structure of the TM in the organ of Corti, and it may play a role in MET [45]. In the adult mouse, otogelin is still produced by the vestibular supporting cells, which suggests a continuous process of otogelin renewal in the OM. In contrast, in the TM, otogelin should be a long-lasting protein since *OTOG* gene has a low expression in the adult cochlear supporting cells [46].

A novel heterozygous missense variant p.Tyr273Asn was found in the *HMX2* gene in a Finnish family with MD affecting a child and his paternal grandfather [47]. The *HMX2* gene encodes a highly conserved protein involved in the inner ear development in mice [48] and zebrafish [49].

The gene encoding the limbic system associated membrane protein (*LSAMP*) was described in two sisters from a consanguineous Iranian Lur family [50]. The homozygous variant p.Tyr273Asn was classified as likely pathogenic and segregated the MD phenotype. The gene LSAMP is a neuronal surface adhesion glycoprotein in cortical and subcortical regions of the limbic system [51], but its function in the inner ear is not known.

*STRC* encodes stereocilin, a protein that interacts with otogelin and otogelin-like to form crowns in the TM attachment to stereocilia tip and horizontal top connectors in mouse cochlear hair cells [45]. A non-consanguineous Swedish-Norwegian family consisting of two brothers and their first cousin with moderate SNHL and a history of episodic vertigo starting before 6 years old was reported [52]. This child onset MD-like phenotype segregated the homozygous nonsense variant p.Gln1343 in the *STRC* gene [52].

Variants in *STRC* gene cause DFNB16B representing at least 10% of cases with AR, non-syndromic SNHL [53]; however, vestibular symptoms are usually missing.

Taken together, *OTOG* is the most common gene found in familial MD, but cellular or animal models are required to demonstrate the pathogenic effect, particularly in variants of unknown significance with compound recessive inheritance.

### Digenic Inheritance in MD

The *MYO7A* gene encodes a motor protein with a key role in the organization of stereocilia in auditory and vestibular hair cells. Rare variants in the *MYO7A* gene may cause AD or AR SNHL accompanied by vestibular dysfunction or retinitis pigmentosa (Usher syndrome type 1B) [54]. Nine rare coding variants in *MYO7A* gene have been reported in familial MD [31]. Two of them (p.Met1? and p.Trp1545) were loss of function variants, leading to a start loss and stop codon in the sequence, respectively, and classified as likely pathogenic (Table 3); however, the rest of the variants were classified as VUS or likely benign. Of note, some of these families showed a second missense variant in the genes *ADGRV1*, *CDH23*, *PCDH15*, *USH1C*, or *SHROOM2* which also segregated the phenotype, leading to the hypothesis of digenic/polygenic inheritance in familial MD associated with protein in the stereocilia links.

**Table 3:** Genes and variants reported in digenic familial MD

**Gene**	**Chr**	**Position**^**a**^	**ID**	**cDNA**	**Protein**	**Variant effect**	**Allelic Frequency**^**b**^		**ACMG classification**	**CADD score**
							**gnomAD**	**Other**		
*MYO7A*	11	77130637	rs782787126	NM_000260.4:c.3G>A	p.Met1^e^	Nonsense^d^	2.02 × 10^-5^	NF(CSVS) 9 × 10^-6 ^(ExAC)	Likely Pathogenic (PVS1, PM2, PP1)	24.0
	11	77159450	rs45629132	NM_000260.4:c.1007G>A	p.Arg336His	Missense	1.61 × 10^-3^	4.9 × 10^-4^(CSVS) 1.15 × 10^-3^ (ExAC)	Uncertain Significance(PP3, BS1)	24.1
	11	77174877	rs781991817	NM_000260.4:c.2057G>A	p.Arg686His	Missense	3.54 × 10^-4^	3.7 × 10^-3^(CSVS) 1.6 × 10^-4^ (ExAC)	Uncertain Significance (PP3, BS1)	29.8
	11	77179874	rs782179888	NM_000260.4:c.2507G>A	p.Arg836His	Missense	4.6 × 10^-5^	NF(CSVS) 2.3 × 10^-4^ (ExAC)	Uncertain Significance (PM2, PP3)	24.5
	11	77180404	rs200454015	NM_000260.4:c.2617C>T	p.Arg873Trp	Missense^d^	8.41 × 10^-4^	2.4 × 10^-4 ^(CSVS)	Uncertain Significance (PP3, BS1)	24.8
	11	77199601	-	NM_000260.4:c.4635G>A	p.Trp1545*	Nonsense	Novel		(PVS1, PM2)Likely Pathogenic	43
	11	77211830	rs41298759	NM_000260.4:c.6247G>A	p.Ala2083Thr	Missense^d^	2.63 × 10^-4^	2.4 × 10^-4 ^(CSVS) 4.16 × 10^-4^ (ExAC)	Uncertain Significance (PP3)	21.8
	11	77214674	rs776881443	NM_000260.4:c.6626G>A	p.Arg2209Gln	Missense	1.97 × 10^-5^	NF (CSVS)	Uncertain Significance (PM2, PP3)	22.8
	11	77214688	rs111033231	NM_000260.4:c.6640G>A	p.Gly2214Ser	Missense	1.74 × 10^-2^	10^-3^(CSVS) 8.85 × 10^-3^ (ExAC)	Likely Benign (PS4, BS2)	10.4
*ADGRV1*	5	90694338	rs201733037	NM_032119.4:c.7582C>T	p.Pro2528Ser	Missense	3.96 × 10^-3^	5 × 10^-3^(CSVS) 4.3 × 10^-3 ^(ExAC)	Likely Benign (BS2, BP4)	22.3
	5	90840606	rs200907244	NM_032119.4:c.16640G>A	p.Arg5547His	Missense^d^	1.91 × 10^-4^	2.5 × 10^-4^(CSVS) 1.02 × 10^-4^ (ExAC)	Uncertain Significance (PM2)	19.5
*CDH23*	10	71732116	rs149073355	NM_022124.6:c.3845A>G	p.Asn1282Ser	Missense	3.33 × 10^-3^	4 × 10^-3^(CSVS) 2.82 × 10^-3 ^(ExAC)	Likely Benign (PM1, BS1, BP6)	23.2
	10	71793440	rs531513127	NM_022124.6:c.6512G>A	p.Arg2176His	Missense	6.57 × 10^-5^	2.5 × 10^-4 ^(CSVS)	Uncertain Significance (PM2, BP4)	20.2
*PCDH15*	10	53822490	rs762526774	NM_033056.4:c.5257C>A	p.Pro1746Thr	Missense	1.91 × 10^-4^	2.5 × 10^-4 ^(CSVS) 2 × 10^-5 ^(ExAC)	Uncertain Significance (PM2, BP4)	9.5
*USH1C*	11	17509546	rs41282932	NM_153676.4:c.1823C>G	p.Pro608Arg	Missense^d^	5.93 × 10^-4^	2.5 × 10^-4 ^(CSVS)	Uncertain Significance (PS1, PM2)	23.4
*SHROOM2*	X	9894539	rs138558321	NM_001649.4:c.631G>A	p.Gly211Ser	Missense^d^	2.32 × 10^-3^	NF(CSVS) 3.52 × 10^-3 ^(ExAC)	Uncertain Significance (PS4, BS1)	16.6

*ACMG* American College of Medical Genetics and Genomics, *CADD* Combined Annotation Dependent Depletion, *CSVS* Collaborative Spanish Variant Server, *DG* digenic inheritance pattern, *ExAC* Exome Aggregation Consortium, *gnomAD* Genome Aggregation Database, *ID* reference Single Nucleotide Polymorphism identifier, *NF* not found

*stop codon

^a^Positions have been updated according to the GRCh38/hg38 reference genome

^b^allelic frequencies reported in the original reports have been updated according to the available information in the last version of the reference database (gnomAD v3.1.2)

^c^incomplete penetrance

^d^multiple inheritance

^e^start loss

### Molecular Hypothesis to Explain Episodic Symptoms in Meniere Disease

The TM may contribute to regulating Ca^2+^ levels around the hair cell stereocilia and MET channel adaptation [55]. Apparently, behind this function are the VFWD domains of α-tectorin and otogelin, which can bind Ca^2+^ ions acting as a reservoir for Ca^2+^ cations [56, 57]. Several constitutive proteins of the OM and TM, including otogelin and α-tectorin, show rare variations that may result in new electrostatic interactions affecting the 3D structure [25. 35]. These changes may affect the formation of the OM or TM or the attachment of these extracellular structures to the hair cells stereocilia [3]. Since α-tectorin functions as a structural organizer on the surface of the supporting cells to establish the layers of the TM, mutations involving the glycosylphosphatidylinositol anchorage sequence will produce a release of α-tectorin into the luminal space and impair the TM self- assembly process [38].

Moreover, proteins involved in the stereocilia links seems to be also involved in the pathophysiology of MD, including myosin VIIa, cadherin-23, protochadherin-15, or adhesion G-protein coupled receptor V1 (ADGRV1) [31].

The stereocilia in the mature outer hair cells have 2 types of links: the tip links, a filamentous protein formation that connect adjacent stereocilia formed by two cadherin-related proteins, cadherin-23, and protochadherin-15, which is linked to the MET channel [33], and the crown-shaped structures located at the tips of the tall stereocilia that form the TM-attachment crown that involve otogelin, otogelin-like, stereocilin [43], tubby protein homolog, and microtubule‑associated protein 1 A [58].

The network of proteins that connects stereocilia, OM, or TM is essential to preserve not only the OM or TM architecture but also the ionic microenvironment in hair cell bundles. The ionic homeostasis of the otolithic and TM could be critical to suppress the innate motility of individual hair cell bundles and focal detachment of these membranes may cause random depolarization of hair cells and explaining the changes in tinnitus loudness or triggering vertigo attacks [59].

With the progression of the disease, a large detachment will lead to an otolithic membrane herniation into the horizontal semicircular canal with dissociation in caloric and head impulse responses [60]. However, further studies in cellular and animal models are needed to confirm this hypothesis.

## Ackowledgements

We would like to thank the patients with MD and their families for their enthusiastic participation.

## References

[1] Perez-Carpena P, Lopez-Escamez JA (2019) Current understanding and clinical management of Meniere’s disease: a systematic review. Semin Neurol 40(1):138–150 10.1055/s-0039-3402065 31887752

[2] Kim SY, Lee CH, Yoo DM, Min C, Choi HG (2022). Association between asthma and meniere’s disease: a nested case-control study. Laryngoscope.

[3] Radtke A, Lempert T, Gresty MA, Brookes GB, Bronstein AM, Neuhauser H (2002). Migraine and Ménière’s disease: is there a link?. Neurology.

[4] Gazquez I, Soto-Varela A, Aran I, Santos S, Batuecas A, Trinidad G et al (2011) High prevalence of systemic autoimmune diseases in patients with Menière’s disease. PLoS ONE 6(10) 10.1371/journal.pone.0026759 PMC3203881 22053211

[5] Gibson WPR (2019). Meniere’s disease. Adv Otorhinolaryngol.

[6] Pyykkö I, Nakashima T, Yoshida T, Zou J, Naganawa S (2013) Menière’s disease: a reappraisal supported by a variable latency of symptoms and the MRI visualisation of endolymphatic hydrops. BMJ Open 3:e001555 10.1136/bmjopen-2012-001555 PMC3586172 23418296

[7] Moleon MDC, Torres-Garcia L, Batuecas-Caletrio A, Castillo-Ledesma N, Gonzalez-Aguado R, Magnoni L et al (2022) A predictive model of bilateral sensorineural hearing loss in Meniere disease using clinical data. Ear and Hearing 43(3):1079–1085 10.1097/AUD.0000000000001169 34799494

[8] Frejo L, Martin-Sanz E, Teggi R, Trinidad G, Soto-Varela A, Santos-Perez S (2017). Extended phenotype and clinical subgroups in unilateral Meniere disease: a cross-sectional study with cluster analysis. Clin Otolaryngol.

[9] Committee on Hearing and Equilibrium guidelines for the diagnosis and evaluation of therapy in Menière’s disease (1995) American Academy of Otolaryngology-Head and Neck Foundation, Inc. Otolaryngol Neck Surg 113(3):181–5 10.1016/S0194-5998(95)70102-8 7675476

[10] Lopez-Escamez JA, Carey J, Chung WH, Goebel JA, Magnusson M, Mandalà M (2015). Diagnostic criteria for Menière’s disease. J Vestib Res Equilib Orientat.

[11] Rauch SD, Merchant SN, Thedinger BA (1989) Meniere’s syndrome and endolymphatic hydrops. Double-blind temporal bone study. Ann Otol Rhinol Laryngol 98(11):873–83 10.1177/000348948909801108 2817679

[12] Merchant SN, Adams JC, Nadol JB (2005). Pathophysiology of Meniere’s syndrome: are symptoms caused by endolymphatic hydrops?. Otol Neurotol Off Publ Am Otol Soc Am Neurotol Soc Eur Acad Otol Neurotol.

[13] Lopez-Escamez JA, Attyé A (2019). Systematic review of magnetic resonance imaging for diagnosis of Meniere disease. J Vestib Res.

[14] Gürkov R, Kantner C, Strupp M, Flatz W, Krause E, Ertl-Wagner B (2014). Endolymphatic hydrops in patients with vestibular migraine and auditory symptoms. Eur Arch Oto-Rhino-Laryngol Off J Eur Fed Oto-Rhino-Laryngol Soc EUFOS Affil Ger Soc Oto-Rhino-Laryngol - Head Neck Surg.

[15] Frejo L, Gallego-Martinez A, Requena T, Martin-Sanz E, Amor-Dorado JC, Soto-Varela A (2018). Proinflammatory cytokines and response to molds in mononuclear cells of patients with Meniere disease. Sci Rep.

[16] Frejo L, Lopez-Escamez JA (2022). Cytokines and inflammation in Meniere disease. Clin Exp Otorhinolaryngol.

[17] Gallego-Martinez A, Requena T, Roman-Naranjo P, Lopez-Escamez JA (2019). Excess of rare missense variants in hearing loss genes in sporadic Meniere disease. Front Genet.

[18] Brown MR (1949) The factor of heredity in labyrinthine deafness and paroxysmal vertigo; Ménière’s syndrome. Ann Otol Rhinol Laryngol 58(3):665–70 10.1177/000348944905800303 15397195

[19] Bernstein JM (1965). Occurrence of episodic vertigo and hearing loss in families. Ann Otol Rhinol Laryngol.

[20] Morrison AW, Bailey MES, Morrison GAJ (2009). Familial Ménière’s disease: clinical and genetic aspects. J Laryngol Otol.

[21] Requena T, Espinosa-Sanchez JM, Cabrera S, Trinidad G, Soto-Varela A, Santos-Perez S (2014). Familial clustering and genetic heterogeneity in Meniere’s disease. Clin Genet.

[22] Lee JM, Kim MJ, Jung J, Kim HJ, Seo YJ, Kim SH (2015). Genetic aspects and clinical characteristics of familial meniere’s disease in a South Korean population. Laryngoscope.

[23] Martín-Sierra C, Gallego-Martinez A, Requena T, Frejo L, Batuecas-Caletrío A, Lopez-Escamez JA (2017). Variable expressivity and genetic heterogeneity involving DPT and SEMA3D genes in autosomal dominant familial Meniere’s disease. Eur J Hum Genet.

[24] Rizk HG, Mehta NK, Qureshi U, Yuen E, Zhang K, Nkrumah Y (2022). Pathogenesis and etiology of Ménière disease: a scoping review of a century of evidence. JAMA Otolaryngol Neck Surg.

[25] Roman-Naranjo P, Gallego-Martinez A, Soto-Varela A, Aran I, Moleon MDC, Espinosa-Sanchez JM (2020). Burden of rare variants in the OTOG gene in familial Meniere’s disease. Ear Hear.

[26] Gallego-Martinez A, Lopez-Escamez JA (2020). Genetic architecture of Meniere’s disease. Hear Res.

[27] Escalera-Balsera A, Roman-Naranjo P, Lopez-Escamez JA (2020). Systematic review of sequencing studies and gene expression profiling in familial Meniere disease. Genes.

[28] Requena T, Cabrera S, Martin-Sierra C, Price SD, Lysakowski A, Lopez-Escamez JA (2015). Identification of two novel mutations in FAM136A and DTNA genes in autosomal-dominant familial Meniere’s disease. Hum Mol Genet.

[29] Mei C, Dong H, Nisenbaum E, Thielhelm T, Nourbakhsh A, Yan D (2021). Genetics and the individualized therapy of vestibular disorders. Front Neurol.

[30] Oza AM, DiStefano MT, Hemphill SE, Cushman BJ, Grant AR, Siegert RK (2018). Expert specification of the ACMG/AMP variant interpretation guidelines for genetic hearing loss. Hum Mutat.

[31] Roman-Naranjo P, Moleon MDC, Aran I, Escalera-Balsera A, Soto-Varela A, Bächinger D (2021). Rare coding variants involving MYO7A and other genes encoding stereocilia link proteins in familial meniere disease. Hear Res.

[32] Ahmed ZM, Riazuddin S, Aye S, Ali RA, Venselaar H, Anwar S (2008). Gene structure and mutant alleles of PCDH15: nonsyndromic deafness DFNB23 and type 1 Usher syndrome. Hum Genet.

[33] Gillespie PG, Müller U (2009). Mechanotransduction by hair cells: models, molecules, and mechanisms. Cell.

[34] Bahloul A, Michel V, Hardelin JP, Nouaille S, Hoos S, Houdusse A (2010). Cadherin-23, myosin VIIa and harmonin, encoded by Usher syndrome type I genes, form a ternary complex and interact with membrane phospholipids. Hum Mol Genet.

[35] Roman-Naranjo P, Parra-Perez AM, Escalera-Balsera A, Soto-Varela A, Gallego-Martinez A, Aran I (2022). Defective α-tectorin may involve tectorial membrane in familial Meniere disease. Clin Transl Med.

[36] Avraham KB, Khalaily L, Noy Y, Kamal L, Koffler-Brill T, Taiber S (2022). The noncoding genome and hearing loss. Hum Genet.

[37] Flook M, Escalera-Balsera A, Gallego-Martinez A, Espinosa-Sanchez JM, Aran I, Soto-Varela A (2021). DNA methylation signature in mononuclear cells and proinflammatory cytokines may define molecular subtypes in sporadic Meniere disease. Biomedicines.

[38] Kim DK, Kim JA, Park J, Niazi A, Almishaal A, Park S (2019) The release of surface-anchored α-tectorin, an apical extracellular matrix protein, mediates tectorial membrane organization. Sci Adv 5(11):eaay6300 10.1126/sciadv.aay6300 PMC6881170 31807709

[39] Robijn SMM, Smits JJ, Sezer K, Huygen PLM, Beynon AJ, van Wijk E (2022). Genotype-phenotype correlations of pathogenic COCH variants in DFNA9: a HuGE systematic review and audiometric meta-analysis. Biomolecules.

[40] Kim BJ, Kim AR, Han KH, Rah YC, Hyun J, Ra BS (2016). Distinct vestibular phenotypes in DFNA9 families with COCH variants. Eur Arch Otorhinolaryngol.

[41] Robertson NG (2006). Cochlin immunostaining of inner ear pathologic deposits and proteomic analysis in DFNA9 deafness and vestibular dysfunction. Hum Mol Genet.

[42] Lien CF, Vlachouli C, Blake DJ, Simons JP, Górecki DC (2004). Differential spatio-temporal expression of alpha-dystrobrevin-1 during mouse development. Gene Expr Patterns.

[43] Requena T, Keder A, Zur Lage P, Albert JT, Jarman AP (2022). A Drosophila model for Meniere’s disease: dystrobrevin is required for support cell function in hearing and proprioception. Front Cell Dev Biol.

[44] Simmler MC, Cohen-Salmon M, El-Amraoui A, Guillaud L, Benichou JC, Petit C (2000). Targeted disruption of otog results in deafness and severe imbalance. Nat Genet.

[45] Avan P, Gal SL, Michel V, Dupont T, Hardelin JP, Petit C (2019). Otogelin, otogelin-like, and stereocilin form links connecting outer hair cell stereocilia to each other and the tectorial membrane. Proc Natl Acad Sci.

[46] El-Amraoui A, Cohen-Salmon M, Petit C, Simmler MC (2001). Spatiotemporal expression of otogelin in the developing and adult mouse inner ear. Hear Res.

[47] Skarp S, Kanervo L, Kotimäki J, Sorri M, Männikkö M, Hietikko E (2019). Whole-exome sequencing suggests multiallelic inheritance for childhood-onset Ménière’s disease. Ann Hum Genet.

[48] Wang W, Lufkin T (2005). Hmx homeobox gene function in inner ear and nervous system cell-type specification and development. Exp Cell Res.

[49] Feng Y, Xu Q (2010). Pivotal role of hmx2 and hmx3 in zebrafish inner ear and lateral line development. Dev Biol.

[50] Mehrjoo Z, Kahrizi K, Mohseni M, Akbari M, Arzhangi S, Jalalvand K (2020). Limbic system associated membrane protein mutation in an Iranian family diagnosed with Ménière’s disease. Arch Iran Med.

[51] Keller F, Rimvall K, Barbe MF, Levitt P (1989). A membrane glycoprotein associated with the limbic system mediates the formation of the septo-hippocampal pathway in vitro. Neuron.

[52] Frykholm C, Klar J, Tomanovic T, Ameur A, Dahl N (2018). Stereocilin gene variants associated with episodic vertigo: expansion of the DFNB16 phenotype. Eur J Hum Genet.

[53] Vona B, Hofrichter MA, Neuner C, Schröder J, Gehrig A, Hennermann JB et al (2015) DFNB16 is a frequent cause of congenital hearing impairment: implementation of STRC mutation analysis in routine diagnostics. Clin Genet 87(1):49–55 10.1111/cge.12332 PMC4302246 26011646

[54] Delmaghani S, El-Amraoui A (2022). The genetic and phenotypic landscapes of Usher syndrome: from disease mechanisms to a new classification. Hum Genet.

[55] Jeng JY, Harasztosi C, Carlton AJ, Corns LF, Marchetta P, Johnson SL (2021). MET currents and otoacoustic emissions from mice with a detached tectorial membrane indicate the extracellular matrix regulates Ca2+ near stereocilia. J Physiol.

[56] Huang RH, Wang Y, Roth R, Yu X, Purvis AR, Heuser JE (2008). Assembly of Weibel-Palade body-like tubules from N-terminal domains of von Willebrand factor. Proc Natl Acad Sci U S A.

[57] Strimbu CE, Prasad S, Hakizimana P, Fridberger A (2019). Control of hearing sensitivity by tectorial membrane calcium. Proc Natl Acad Sci U S A.

[58] Youn SY, Min H, Jeong SR, Lee J, Moon SJ, Bok J (2022). Microtubule-associated protein 1 A and tubby act independently in regulating the localization of stereocilin to the tips of inner ear hair cell stereocilia. Mol Brain.

[59] Senofsky N, Faber J, Bozovic D (2022). Vestibular drop attacks and Meniere’s disease as results of otolithic membrane damage-a numerical model. JARO - J Assoc Res Otolaryngol.

[60] Shen LL, Andresen NS, Chari DA, Pogson JM, Lauer AM, Rabbitt RD (2022). Otolith membrane herniation, not semicircular canal duct dilation, is associated with decreased caloric responses in Ménière’s disease. JARO - J Assoc Res Otolaryngol.

[61] Farhadi M, Razmara E, Balali M, Hajabbas Farshchi Y, Falah M (2021). How Transmembrane Inner Ear (TMIE) plays role in the auditory system: a mystery to us. J Cell Mol Med.

[62] Andrade LR, Salles FT, Grati M, Manor U, Kachar B (2016). Tectorins crosslink type II collagen fibrils and connect the tectorial membrane to the spiral limbus. J Struct Biol.

[63] Peng AW, Salles FT, Pan B, Ricci AJ (2011). Integrating the biophysical and molecular mechanisms of auditory hair cell mechanotransduction. Nat Commun.

[64] Richardson GP, de Monvel JB, Petit C (2011). How the genetics of deafness illuminates auditory physiology. Annu Rev Physiol.

